# Assessing Drivers of Medication Management Hassles among Care Partners of Older Adults

**DOI:** 10.1093/geroni/igaf122.3709

**Published:** 2025-12-31

**Authors:** Sohee Kim, Te-Lien Ku, Shu-Ying Wu, Yen-Ming Huang

**Affiliations:** University of Wisconsin - Madison, Madison, Wisconsin, United States; University of Wisconsin-Madison, Madison, Wisconsin, United States; Sijhih Cathay General Hospital, New Taipei City, New Taipei, Taiwan (Republic of China); National Taiwan University, Taipei City, Taipei, Taiwan (Republic of China)

## Abstract

Care Partners (CPs)—such as family members or friends—play a critical role in assisting older adults with multiple chronic conditions in managing complex medication regimens. However, CPs frequently report feeling burdened by these responsibilities. This study aimed to examine factors associated with medication management hassles reported by CPs. We surveyed 138 CPs in Northern Taiwan who assisted with or were responsible for managing medications for family members aged 65 or older. Data collection included demographics of both CPs and care recipients, care recipient health status, CPs’ caregiving experiences, and perceived stressors related to medication management—measured using the Traditional Chinese version of Family Caregiver Medication Administration Hassles Scale (FCMAHS-TC). Data analysis included LASSO (Least Absolute Shrinkage and Selection Operator) regression to select key predicting variables, followed by multivariable linear regression to examine associations with FCMAHS-TC scores. Time spent on medication management (β = 0.18, p = 0.002) and strained (difficult or uneasy) CPs–recipient interactions (β = 7.84, p = 0.040) were significantly associated with higher hassle scores. Housekeeping CPs showed significantly lower hassles than those in full-time working CPs (β = -9.74, p = 0.035). Future research should further explore the contextual and relational mechanisms underlying the associations between identified factors and CPs’ perceived medication management hassles, informing the development of interventions that better support CPs in navigating their medication responsibilities.

